# The persuasive potential of AI-paraphrased information at scale

**DOI:** 10.1093/pnasnexus/pgaf207

**Published:** 2025-07-22

**Authors:** Saloni Dash, Yiwei Xu, Madeline Jalbert, Emma S Spiro

**Affiliations:** Information School, University of Washington, 1851 NE Grant L, Seattle, WA 98105, USA; Information School, University of Washington, 1851 NE Grant L, Seattle, WA 98105, USA; Information School, University of Washington, 1851 NE Grant L, Seattle, WA 98105, USA; Information School, University of Washington, 1851 NE Grant L, Seattle, WA 98105, USA

**Keywords:** generative AI, persuasion, cognitive heuristics, illusory truth effect, information campaigns

## Abstract

In this article, we study how AI-paraphrased messages have the potential to amplify the persuasive impact and scale of information campaigns. Building from social and cognitive theories on repetition and information processing, we model how *CopyPasta*—a common repetition tactic leveraged by information campaigns—can be enhanced using large language models. We first extract CopyPasta from two prominent disinformation campaigns in the United States and use ChatGPT to paraphrase the original message to generate *AIPasta*. We then validate that AIPasta is lexically diverse in comparison to CopyPasta while retaining the semantics of the original message using natural language processing metrics. In a preregistered experiment comparing the persuasive potential of CopyPasta and AIPasta (*N* = 1,200), we find that AIPasta (but not CopyPasta) is effective at increasing perceptions of consensus in the broad false narrative of the campaign while maintaining similar levels of sharing intent with respect to Control (CopyPasta reduces such intent). Additionally, AIPasta (vs. Control) increases belief in the exact false claim of the campaign, depending on political orientation. However, across most outcomes, we find little evidence of significant persuasive differences between AIPasta and CopyPasta. Nonetheless, current state-of-the-art AI-text detectors fail to detect AIPasta, opening the door for these operations to scale successfully. As AI-enabled information operations become more prominent, we anticipate a shift from traditional CopyPasta to AIPasta, which presents significant challenges for detection and mitigation.

Significance Statement Advancements in generative AI have fueled worries about its misuse for enhancing harmful information operations by lowering the cost of producing persuasive, high-quality arguments and/or microtargeting vulnerable subpopulations. This article demonstrates how simple tactics like paraphrasing messages using AI to produce nonverbatim, repetitive messaging can be effective and hard to detect, exposing new information vulnerabilities that merit continued study.

## Introduction

The impact of generative AI tools like GPT4 and DALL-E on information campaigns has garnered significant attention and debate from experts and media alike (1–4). A 2024 World Economic Forum (WEF) report (5) highlighted the role of synthetic content produced by AI in magnifying the risks of manipulated and falsified information, declaring it the greatest short-term global threat. Recent studies exploring the persuasive effects of AI-driven mis/disinformation (6, 7) suggest that AI-generated content can be more compelling to consumers due to its ability to produce high-quality arguments tailored to groups and individuals (8–12).

A potential information threat that has received limited attention is the use of generative AI to boost the effectiveness of repetitive messaging across multiple social network sites and accounts (13–15). Information repetition tactics exploit the *“illusory truth effect”*—a well-established phenomenon that shows that repeating information increases its truth perceptions (16, 17). *CopyPasta* campaigns, where a single message manually crafted by adversarial actors is shared repeatedly at scale, are a common example of repetition-based information campaigns. *CopyPasta* campaigns have been frequently used to inflate the prominence of and exposure to topics online, and prior work has demonstrated their role in sharing false and misleading narratives or claims. For example, CopyPasta campaigns were used within #StopTheSteal rhetoric on X (formerly known as Twitter) (18, 19) to amplify fringe ideologies leading up to the US Capitol insurrection on 2021 January 6th. Similarly, during the COVID-19 pandemic, CopyPasta campaigns were observed surrounding the release of the short video called “Plandemic” which falsely claimed a group of societal elites was using the virus and a potential vaccine to profit and gain power (19, 20). The prevalence of CopyPasta in today’s information environment has prompted some platforms to respond with mitigation attempts; X (formerly Twitter) previously announced that it would suppress low-quality posts that contained “*similar or duplicate content*.”

Although CopyPasta is effective at flooding platforms with propaganda messaging, the persuasive impact of such campaigns could be limited as repetitive posts with identical text may trigger psychological reactance in platform users (21). With the rapid development and adoption of generative AI, we anticipate that CopyPasta campaigns can be enhanced using large language models (LLMs) to generate AI-paraphrased information or repetitive messages that are not identical—what we call *AIPasta*. Building on social and cognitive theories of repetition and information processing, we argue that AIPasta campaigns have the potential to amplify the persuasive impact and scale of information operations, including disinformation.

Despite empirical work on CopyPasta campaigns and more recent concerns about the role of AI in this domain, to our knowledge, the effects of repeated exposure to campaign-related social media messages on belief and consensus have never been systematically tested. Thus, this research serves as an initial investigation of how exposure to repetitive campaign messages impacts downstream assessments of campaign-related claims as well as a test of whether AIPasta may have persuasive advantages beyond CopyPasta. We describe potential underlying mechanisms below.

### Theoretical background

When evaluating information, individuals often rely on a fast and intuitive strategy of processing (also referred to as “heuristic” or “system 1” processing) over a more slow and analytical approach (also referred to as “systematic” or “system 2” processing; (22, 23)). How easy or difficult information feels to process (its *processing fluency*) plays a key role in guiding intuitive judgments. When information feels easier to process, it is also judged to be more true and widely accepted. Often, processing ease can be a valid cue for truth or consensus. For example, information may feel easy to process because it has been encountered from a variety of credible sources. Processing ease may also be a fallible cue for truth; for example, if information feels easy to process because it was repeated from a single source as part of a persuasive attempt (for reviews, see Refs. (22, 24)).

Prior work has found that the mere repetition of information can increase perceptions of its truth, i.e. the “illusory truth effect” (16, 17). This effect occurs both with verbatim repetition and nonverbatim repetition (25, 26), including in a social media context (27). More recent work has also demonstrated that the mere repetition of information can also increase perceptions of its consensus—an “illusory consensus effect” (28). This effect has been found both when different sources share nonverbatim information as well as when one source repeats the same information (27). Perceiving social consensus around claims can further have downstream effects on belief and behavior (29), ranging from changed attitudes towards climate change (30) to increased vaccination uptake (31). Indeed, triangulation of sources has been a core element of information literacy curricula for many years (32, 33).

Building on this prior work, we hypothesize that exposure to both CopyPasta and AIPasta will increase the perceived truth and perceived consensus of messages related to the targeted information campaign. However, we also expect that AIPasta—which relies on nonverbatim repetition—may have a greater persuasive impact on outcomes like perceptions of social consensus. Individuals may identify the verbatim messages used by CopyPasta as originating from the same source, making them less impactful compared to messages perceived to be independent of each other (e.g. (29)). Typically, the size of the truth effect becomes larger with each additional repetition (34). However, when additional repetitions result in a message being perceived as a persuasive attempt, this may result in reactance and additional repetitions now starting to decrease (rather than further increase) a message’s credibility (21). If the nonverbatim repetition from AIPasta makes it less likely to be perceived as a coordinated persuasive effort, this may decrease reactance and increase its persuasive effects.

In addition to the potential of CopyPasta and AIPasta to influence belief and perceived consensus, we also explore whether exposure increases how likely individuals are to share related campaign posts they encounter, as this would further contribute to campaign spread. Some investigations have found that repetition can increase *sharing intention* (35), although also see Ref. (36), which in turn can amplify the spread of mis/disinformation online (37). This effect has been proposed to occur because repetition first increases perceived truth, which then increases sharing intent (37). Thus, exposure to both CopyPasta and AIPasta may later increase intent to share related posts, but if AIPasta increases the perceived truth of messages more than CopyPasta, it may also result in greater sharing intent.

### The present research

To strengthen the ecological validity of our study, we model the potential threat of AI-paraphrased information (AIPasta), by sourcing false claims from two empirical, hashtag-based disinformation campaigns mentioned previously: #StopTheSteal and #Plandemic. After collecting posts containing the hashtags #StopTheSteal and #Plandemic, we extract CopyPasta messages by using a community detection algorithm applied to a similarity graph derived from high-dimensional vector embeddings of the campaign messages (refer to Materials and methods for details). We then use gpt-3.5-turbo (38) to paraphrase the claims in the posts to generate nonverbatim repetitive messages, which we call *AIPasta*. We validate the constructed AIPasta through natural language processing (NLP) metrics of *semantic similarity* (how similar the meaning of two statements are) and *lexical diversity* (how varied the vocabulary is), and find—matching our expectation—that AIPasta is more lexically diverse than CopyPasta while retaining semantic similarity.

We then conduct a preregistered experiment to evaluate the impact of exposure to AIPasta and CopyPasta campaigns. Participants viewed posts containing either AIPasta messaging, CopyPasta messaging, or control messaging and, for each campaign, answered a number of questions relevant to persuasive outcomes including perceived truth, perceptions of social consensus, and sharing likelihood. The Materials and methods section contains preregistered hypotheses, stimuli, and additional details of the study design. Preregistration can be found at OSF.^a^

Broadly, we find that AIPasta (but not CopyPasta) is effective at increasing perceptions of consensus in the broad false narratives of the campaign. In addition, exposure to AIPasta does not change levels of sharing intent compared to a control condition, while exposure to CopyPasta *reduces* sharing intent compared to the control. Additionally, our exploratory analyses indicate that AIPasta (vs. Control) increases belief in the exact campaign claims depending on political orientation. However, across most outcomes, we find little evidence of significant persuasive differences between AIPasta and CopyPasta. Subsequently, we explore whether state-of-the-art AI-text generators can be used to mitigate the impact of these campaigns and find that they perform poorly at identifying AIPasta as AI-generated content, potentially enabling these campaigns to scale unchecked. Therefore, as AI-enabled disinformation operations become more prominent, we anticipate that CopyPasta campaigns may be replaced by AIPasta campaigns. At the time of writing this article, reports suggest that ChatGPT is already being used to generate and paraphrase political propaganda in Rwanda (39), Russia, China, and Iran ^b^—and we expect a growing recognition of these applications.

Our work makes several contributions: (i) to our knowledge, this is the first study to systematically evaluate the effects of repeated exposure to CopyPasta, and subsequently AIPasta, on downstream assessments of social media campaign-related messaging, (ii) conceptually, we bridge the gap between social and cognitive theories on repetition and heuristic processing and empirical studies on disinformation tactics, (iii) we show that AI-paraphrased nonverbatim repetition (AIPasta) has distinct persuasive impacts not observed with verbatim repetition (CopyPasta), and (iv) we show how AI-text detectors fail to recognize AI-paraphrased social media messages as AI-generated.

## Study 1 (AIPasta validation and persuasion)

### Validating AIPasta as nonverbatim repetition

**Fig. 1.**
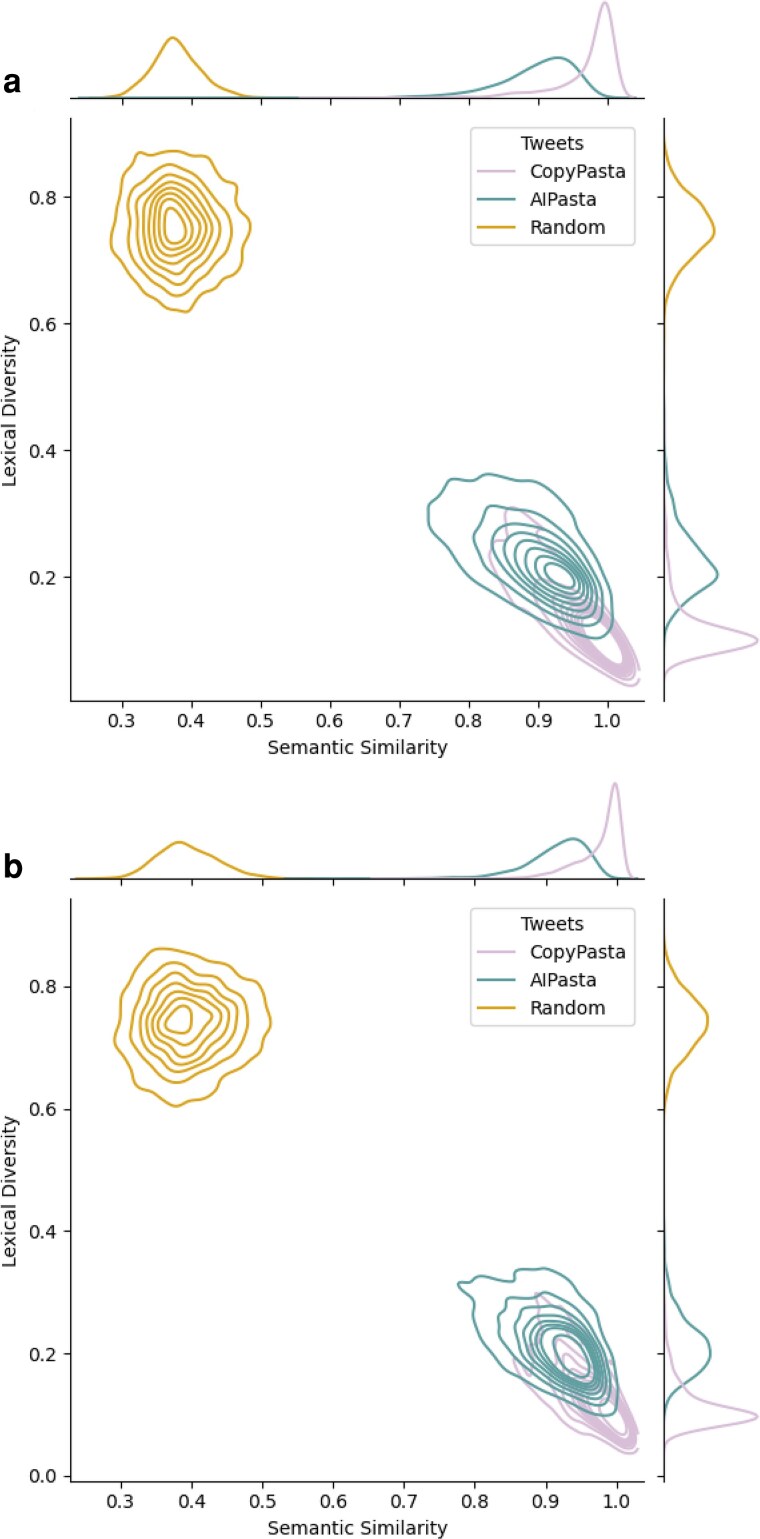
Metrics of lexical diversity and sematic similarity across conditions. AIPasta is observed to be more lexically diverse, while similar semantically, to CopyPasta. Both CopyPasta and AIPasta are distinctly different from random posts. a) #StopTheSteal Metrics. b) #Plandemic Metrics.

In order to systematically evaluate the effects of exposure to both verbatim (CopyPasta) and nonverbatim (AIPasta) repetition, we first need to validate that AIPasta is indeed a form of nonverbatim repetition. To validate our approach, we compare content (i.e. social media posts used later as experimental stimuli) along two dimensions using NLP metrics: (i) *semantic similarity*—how closely related the meaning of two pieces of text are and (ii) *lexical diversity*—the range of vocabulary in a given text (refer to Materials and methods for details). We find that AIPasta increases the lexical diversity of the original CopyPasta by 44% on average, while slightly decreasing the semantic similarity by roughly 7% across both campaigns (#StopTheSteal and #Plandemic), as seen in Figure 1. This indicates that while the AIPasta becomes more varied and richer in vocabulary, it largely maintains the original meaning from CopyPasta. Matching our expectation, and in line with the known strengths of LLMs, AIPasta is easy to produce and demonstrates characteristics that offer potential strategic advantages in information operations (such as the anticipated difficulty for machines and humans to recognize the content as verbatim or duplicate from a single source).

### Effects on perceived truth

We first investigate the effects of AIPasta and CopyPasta on perceived truth across three measures of belief: belief in the exact claim viewed during stimuli exposure, belief in a claim related to the broader false narrative, and belief in the broader disinformation narrative itself (refer to Materials and methods for more details).

**Fig. 2.**
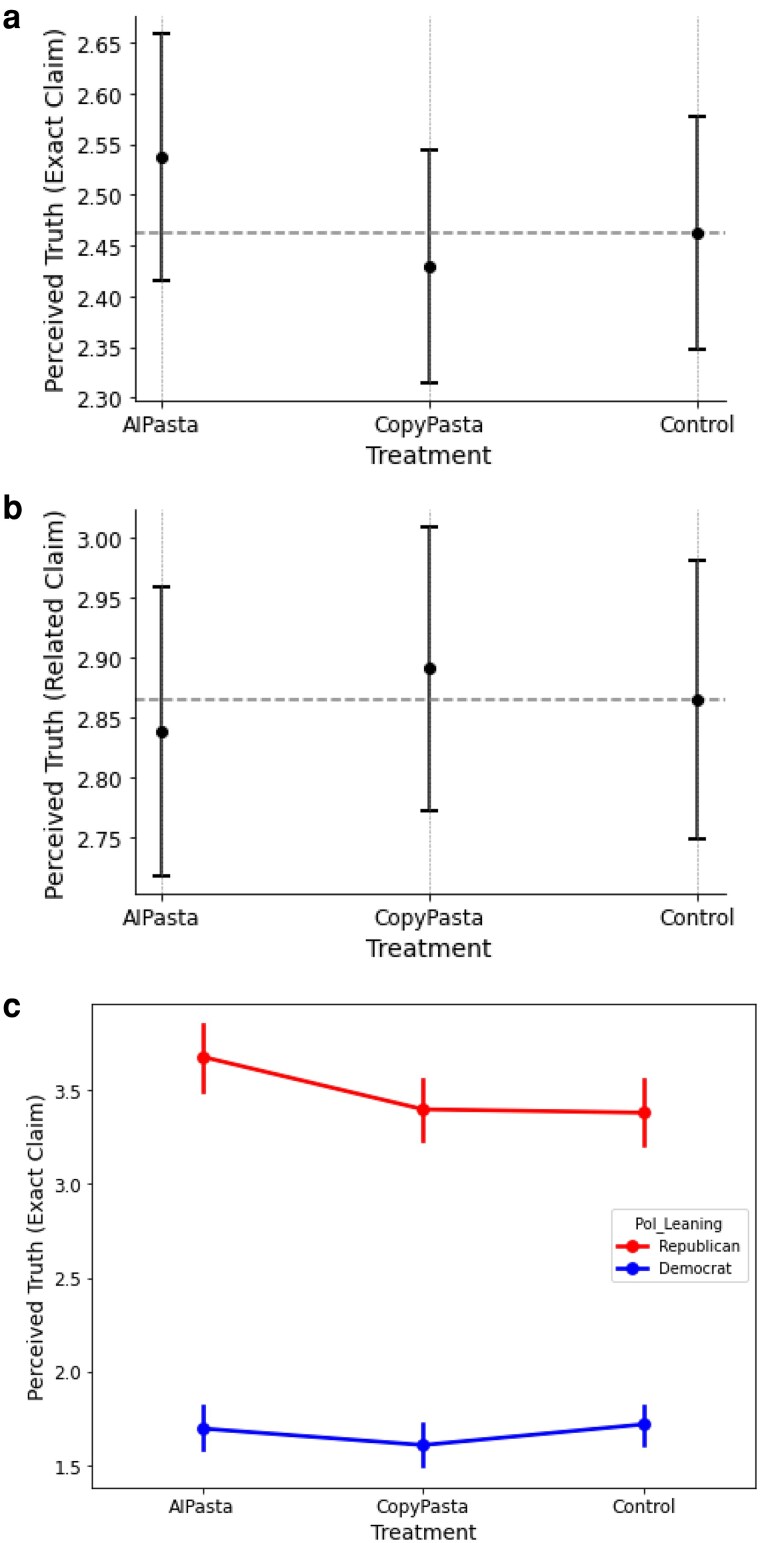
Perceived truth across conditions. Participants exposed to AIPasta were marginally more likely to believe the exact false claim as compared to Control (a), Participants exposed to CopyPasta were marginally more likely to believe the related false narrative as compared to Control (b), and Republican participants in the AIPasta condition were significantly more likely to believe the exact false claim (vs. Control) (c). a) Perceived Truth (Exact Claim). b) Perceived Truth (Related Narrative). c) Perceived Truth (Exact Claim) By Political Party.

Surprisingly, there is no significant effect of AIPasta (vs. Control) on any of these three outcome variables (exact claim b=0.133, CI=[−0.021,0.287], P=0.091; broad claim b=0.020, CI=[−0.112,0.151], P=0.770; related claim b=0.049, CI=[−0.081,0.179], P=0.460), although the effect of exact claim is marginally significant in the predicted direction. We also fail to observe a significant effect of repetition of CopyPasta (vs. Control) on any of the three measures (exact post b=0.006, CI=[−0.152,0.164], P=0.939; broad claim b=0.079, CI=[−0.052,0.211], P=0.237; related claim, b=0.129, CI=[0.000,0.259], P=0.051). Although there is a marginal effect of repetition in the predicted direction for a couple of comparisons (exact claims for AIPasta and the related claim for CopyPasta, see Fig. 2a and b), taken as a whole, our results fail to support H1 (refer to Materials and methods for the hypotheses).

Next, we investigate whether participants who view AIPasta have higher levels of perceived truth for each of the three measures (H2). In each, the difference between the AIPasta and CopyPasta conditions fail to reach significance, with participants in the AIPasta condition not significantly differing from participants in the CopyPasta condition on perceived truth for exact posts previously seen (b=0.109, CI=[−0.019,0.237], P=0.095) a claim related to the broader false narrative (b=−0.060, CI=[−0.192,0.072], P=0.375), or in the broader disinformation narrative itself (b=−0.080, CI=[−0.211,0.050], P=0.227).

The failure to observe significant effects in these conditions may, in part, reflect the fact that participants were at low levels of belief in these claims (2.48±0.07 for exact claims, 2.86±0.07 for related claims, and 2.66±0.07 for broader narrative claim; ratings made on a six-point scale; 95% CI reported), and illusory truth effects are strongest among ambiguous information where veracity may be unknown at the time of exposure (e.g. (40)).

Although not originally hypothesized in the preregistered design, we conduct exploratory analyses to examine whether (i) familiarity with the false claims and (ii) partisanship moderate observed effects. Including familiarity aims to address the concern that participants with prior exposure to similar campaign messages might be more familiar with these false claims, potentially making them less susceptible to persuasion through additional exposure. Additionally, since the topics investigated in this study are highly politicized and the issue positions have been shown to appeal more to Republicans (41, 42), we suspected that partisanship may play a role in moderating belief. While we analyze the moderation effects of both familiarity and partisanship for this and all subsequent outcome variables, we report only statistically significant moderation effects.

Indeed, as shown in Figure 2c, levels of belief in the exact claims in the control condition are higher for Republicans (3.93±0.16; 95% CI) than for Democrats (2.01±0.12; 95% CI); t(663.03)=19.02, P<0.001. Therefore, we conduct an exploratory analysis of whether and how partisanship moderates belief. We indeed find that Republican participants show a greater increase in belief in the exact false claim when exposed to AIPasta (vs. Control) compared to Democrat participants (b=0.304, CI=[0.005,0.603], P=0.046).

### Perceived persuasion

**Fig. 3.**
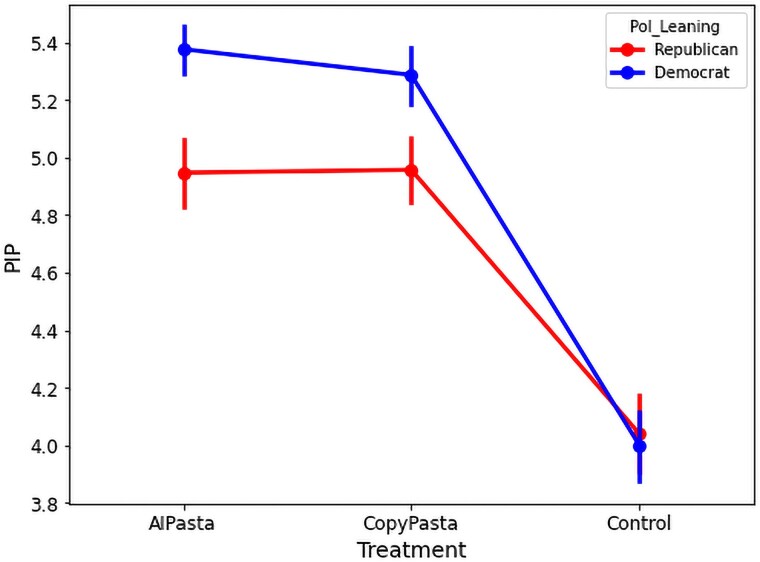
Perceived intent to persuade across conditions moderated by political party. Republican participants exposed to AIPasta or CopyPasta are significantly more likely to feel like they are being persuaded (vs. Control).

Next, we hypothesized that participants exposed to AIPasta posts would give lower ratings when asked whether the authors of the posts were trying to convince them of their views, compared to the participants exposed to CopyPasta posts, but did not find this to be the case (b=0.070, CI=[−0.030,0.171], P=0.070) failing to support H3. Among Republican participants, however, those exposed to AIPasta (b=−0.438, CI=[−0.640,−0.236], P<0.001) or CopyPasta (b=−0.396, CI=[−0.596,−0.195], P<0.001) were significantly less likely to feel like they are being persuaded (vs. Control), as seen in Fig. 3.

### Perceived social consensus

**Fig. 4.**
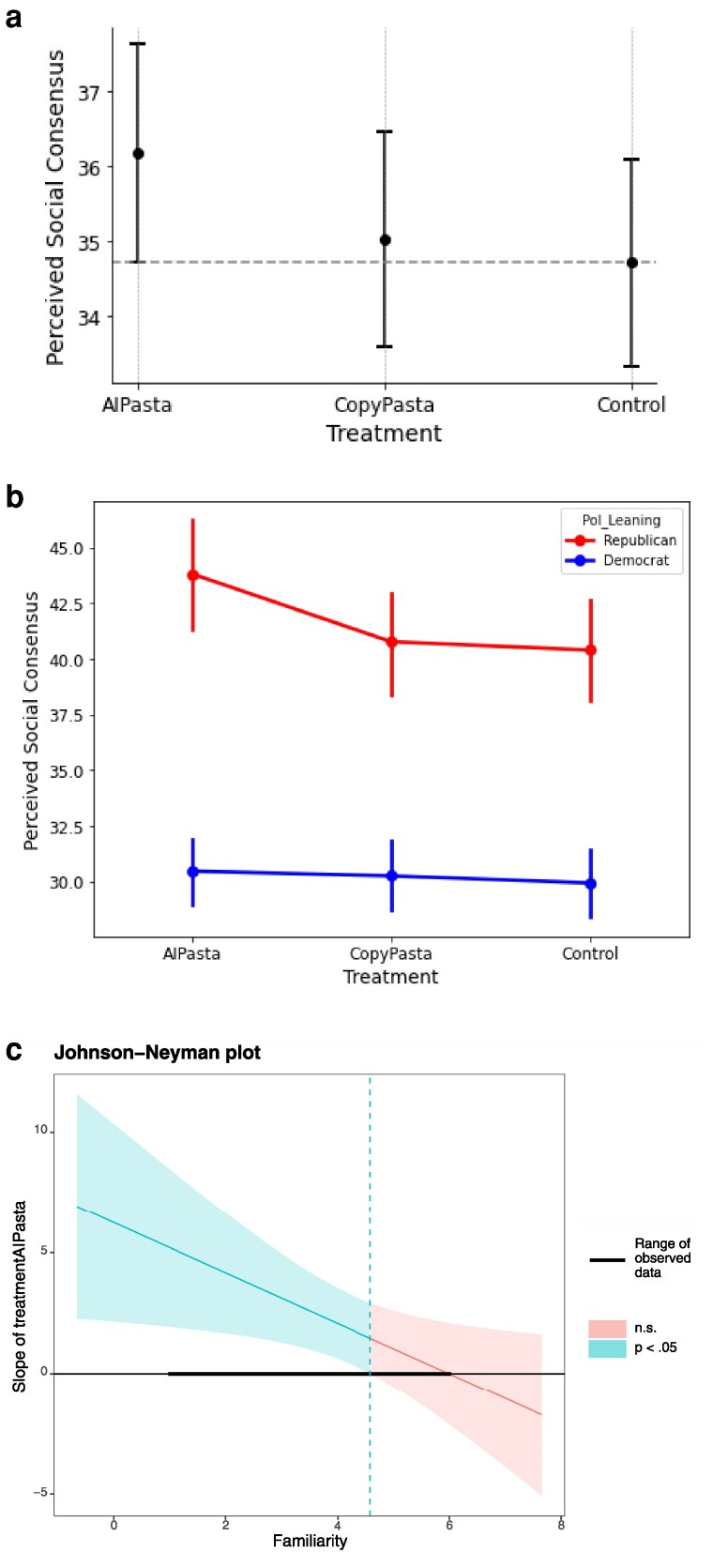
Perceived social consensus across conditions. Participants exposed to AIPasta perceive greater social consensus for the broad false narrative claim as compared to Control. 95% CIs are displayed (a). Republican participants show a greater increase in consensus on the broad false narrative claim after exposure to AIPasta (vs. Control) compared to Democrat participants (b). Participants less familiar with the targeted topic (cutoff point = 4.4) showed a greater increase in perceived consensus on the broad false claims after exposure to AIPasta (vs. Control) compared with participants who were more familiar with the issue (c). a) Perceived Social Consensus. b) Perceived Social Consensus (By Political Party). c) Johnson–Neyman Plot (By Issue Familiarity).

Participants exposed to AIPasta judged that there was greater consensus for the broad false narrative claim than those in the Control condition (b=1.727, CI=[0.246,3.208], P=0.022). This effect, however, is not significant for the CopyPasta condition compared to Control (b=1.200, CI=[−0.281,2.680], P=0.112). Thus, H4 was only partially supported. Additionally, the differences in perceived consensus between CopyPasta and AIPasta did not reach significance (b=0.527, CI=[−0.960,2.015], P=0.487), lending support to H5b (that consensus ratings would be similar for participants exposed to AIPasta and CopyPasta conditions) over H5a (that consensus ratings would be greater for those exposed to AIPasta over CopyPasta (see Fig. 4a).

To follow up on these results, we conduct exploratory analyses investigating whether these effects are moderated by familiarity and political orientation. Political orientation moderates the perception of social consensus(see Fig. 4b), with Republican participants showing a greater increase in consensus on the broad false narrative claim after exposure to AIPasta (vs. Control) compared to Democrat participants (b=3.395, CI=[0.420,6.370], P=0.026).

Figure 4c shows that participants less familiar with the targeted topic (cutoff point = 4.4), showed a greater increase in consensus on the broad false claims after exposure to AIPasta (vs. Control) compared with participants who were more familiar (b=−1.043, CI=[−1.94,−0.147], P=0.023).

### Sharing intention

Surprisingly, participants exposed to CopyPasta report a significantly lower likelihood of sharing a randomly selected CopyPasta post among the stimuli compared to those in the Control condition (b=−0.187, CI=[−0.299,−0.075], P<0.001), rejecting H8. However, this effect did not reach significance when comparing the reported likelihood of sharing an AIPasta post for participants exposed to AIPasta posts compared to those in the Control condition (b=−0.055, CI=[−0.168,0.058], P=0.341).

**Fig. 5.**
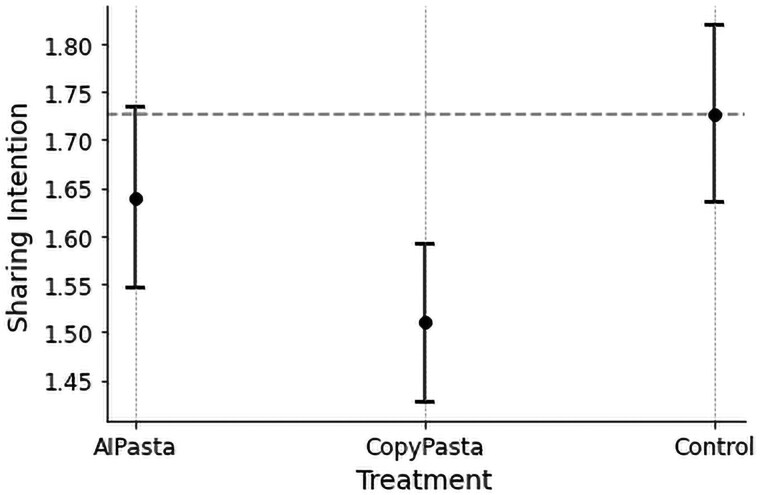
Sharing intention across conditions. Participants exposed to CopyPasta report a significantly lower likelihood of sharing a randomly selected CopyPasta post among the stimuli compared to those in the Control condition, and those exposed to AIPasta were marginally significantly more likely to share the messages as compared to CopyPasta.

When comparing sharing intent for the AIPasta vs. CopyPasta conditions, participants in the AIPasta condition indicated having a directionally higher sharing intent, but this effect only reached marginal significance (b=0.083, CI=[−0.008,0.175], P=0.075), failing to support H9 (see Fig. 5 for an overview).

## Study 2 (AIPasta detection)

As discussed previously, the AIPasta produced in this study is more lexically diverse than CopyPasta. This has downstream consequences for detection; traditional CopyPasta detection algorithms on platforms like X (formerly Twitter)^c^ rely on similarity metrics to suppress the spread of repetitive messaging and may be unable to detect AIPasta due to its increased lexical diversity.

An alternative strategy for detecting AIPasta could involve using off-the-shelf AI-text detectors. To evaluate the detection performance on our dataset, we use a wide array of AI-text detectors (refer to Supplementary Information for more details) to classify our stimuli as AI-generated and human-written, respectively. As seen in Table 1, the AI-text detectors perform poorly at differentiating between AIPasta and CopyPasta (most detectors do marginally better than random, i.e. 50% for a binary classification task), demonstrating that AIPasta is indistinguishable from human-written text according to existing detectors. This is consistent with prior studies (43–45) that have shown that traditional AI-text detectors have reduced performance on AI-paraphrased text (both from human-written and AI-generated) as compared to AI-generated text.

**Table 1.** AIPasta detection (#StopTheSteal).

Detector	CopyPasta recall (%)	AIPasta recall (%)	Avg recall (%)
GLTR (48)	49.01	52.92	50.97
MAGE (44)	50.22	62.41	56.32
RoBERTa-MPU (49)	98.85	4.79	51.82
PPL (47)	96.65	30.43	63.54
OpenAI (50)	64.13	44.97	54.55

Off-the-shelf AI-generated text detectors perform poorly at detecting AIPasta as AI-generated. Most models perform only marginally better than random. We observe similar performances for #Plandemic AIPasta (refer to the Supplementary Information for more details).

To further probe what factors might lead to the low performance of these models, we use a common metric called *perplexity* (46), which measures a language model’s confidence in predicting the next token of a given sequence of text. Previous studies have shown that AI-generated texts are typically associated with lower perplexity scores than human-written text (47). However, little is known about whether patterns observed for AI-generated texts hold for AI-paraphrased texts. We use the open-source GPT-2-XL model to compute perplexity scores for AIPasta and CopyPasta posts by using the Python Huggingface library^d^.

**Fig. 6.**
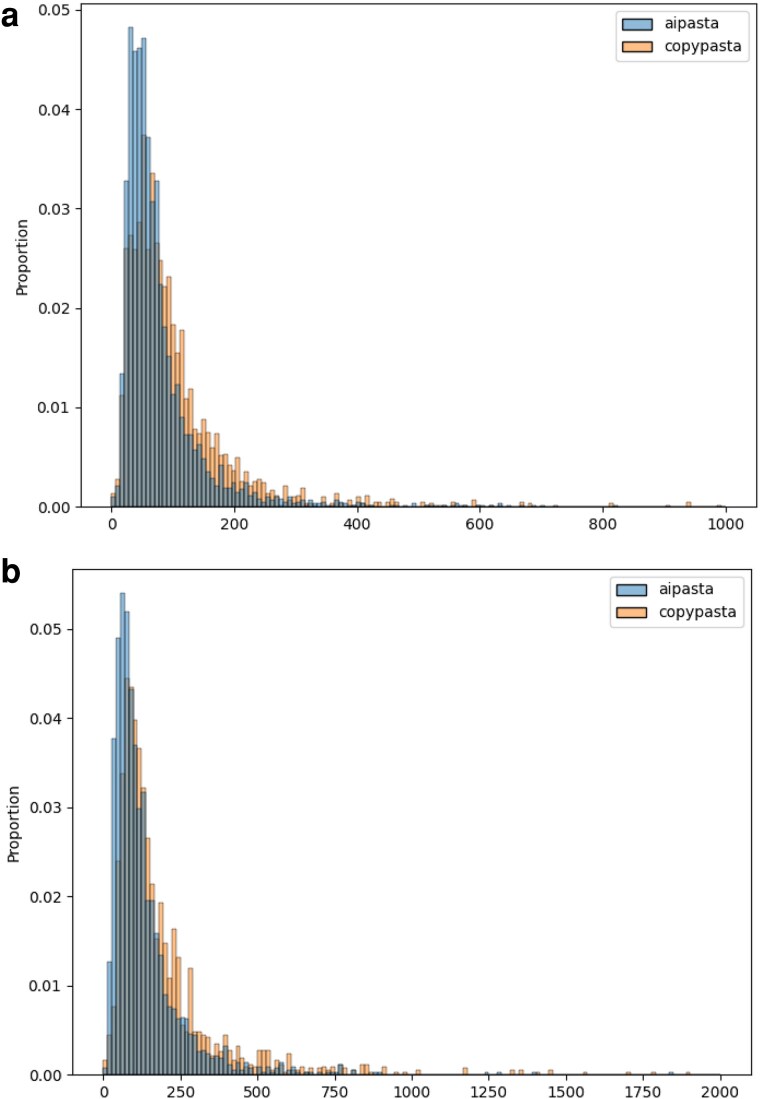
Distribution of perplexity scores. Perplexity score distributions for AIPasta and CopyPasta almost completely overlap. a) #StopTheSteal. b) #Plandemic.

As shown in Fig. 6, we find that the perplexity score distributions for AIPasta and CopyPasta almost completely overlap, highlighting a potential vulnerability in AI-text detectors that primarily rely on perplexity-based metrics for classification. Additionally, we investigate whether AIPasta has any differentiating linguistic characteristics compared to CopyPasta. We examine a variety of linguistic signatures, including sentiment polarity, named-entity tags, and part-of-speech tags (details in Supplementary Information), and find no discerning patterns that can reliably distinguish AIPasta from CopyPasta (see Supplementary Information), underscoring the challenges in detecting AI-paraphrased content through these metrics alone.

## Discussion

We investigate if and how AI-paraphrased social media messages may impact the persuasive potential and scale of information campaigns. First, using an LLM to paraphrase CopyPasta messages from two prominent US-based disinformation campaigns—#StopTheSteal and #Plandemic—to generate AIPasta; we validate that AIPasta retains the semantics of the original message while enhancing its lexical diversity. Next, in a preregistered experiment, we find that exposure to AIPasta (but not CopyPasta) increases perceptions of consensus in the disinformation campaign false claim. AIPasta differentially increases belief in false claims depending on political partisanship and prior familiarity. While exposure to CopyPasta messaging (vs. Control) decreased the reported likelihood of sharing (perhaps due to reactance to viewing the repetition message or perceived lack of novelty of the message), exposure to AIPasta messaging did not have this effect. However, contrary to our initial hypotheses, the experiment did not demonstrate any significant differences between AIPasta and CopyPasta conditions for any of the three belief outcomes (belief in an exact post, the broad claim, and the related claim) when looking across all participants. Taken together, these findings indicate that, unlike CopyPasta, exposure to AIPasta may increase perceptions of consensus in a false narrative while maintaining similar levels of sharing, two key components in effective disinformation campaign tactics. In addition, current state-of-the-art AI-text detectors fail to identify AIPasta, opening the door for these operations to scale successfully, avoiding platform moderation or removal.

An important consideration is that our choice of design may have limited our ability to observe the impact of repetition. In our experiment, unlike in typically illusory truth studies, participants were only making ratings related to the repeated messaging for one campaign and for different messaging for another campaign, and these new and repeated ratings were separated by an exposure phase. In typical illusory truth effect studies, participants judge a mix of new and repeated items during the same judgment phase. Importantly, fluency effects—such as the illusory truth effect—emerge when individuals experience a change in fluency (such as when going from a new item to a repeated item) or when fluency experiences differ from what is expected (51). Indeed, illusory truth effects may not occur in cases where individuals are only judging repeated information rather than a mix of repeated and new information (e.g. (52)). This methodological design may have thus limited our ability to observe truth effects that would have been observed if new items had been mixed with repetitive messages.

However, our exploratory results point toward a more nuanced picture, with some of the persuasive effects of AIPasta varying across individual differences in political partisanship and prior familiarity with the narrative referred to in the social media post. Specifically, we found that Republican (vs. Democrat) respondents had a larger increase in belief in an exact post for AIPasta (vs. Control).

We had predicted that viewing CopyPasta and AIPasta would increase the intent of sharing of related claims compared to a control condition, and that this intent to share may be higher in the AIPasta vs. CopyPasta condition. However, we found that participants’ intent to share campaign posts was significantly lower after viewing CopyPasta compared to a control condition, and did not significantly differ after seeing AIPasta compared to after seeing control posts. And, while intent to share was directionally higher in the AIPasta condition compared to the CopyPasta condition, this effect only reached marginal significance. It is also important to take a broader lens when interpreting what these findings may mean for the total sharing (and thus spread) of these posts. In our study, we only measured sharing intent for a single post. In the real world, individuals have the opportunity to decide to share each post they see. Since we observe a decline in sharing intent after previous exposure to CopyPasta but not after exposure to AIPasta, it raises the concerning possibility that the total spread of AIPasta posts would be greater and decline more slowly over time.

Alarmingly, AIPasta evades machine (and likely human) detection, making it more likely to spread unchecked, while also having distinct persuasive advantages (vs. Control)—specifically, higher perceived social consensus for broad claims (among all participants) and greater belief in exact claims (only among Republican participants). This combination is likely to amplify its effectiveness compared to CopyPasta.

Recognition and correction of false and/or misleading information are key factors in mitigating potential harms; this study suggests that AIPasta targets these vulnerabilities. When encountering information, people tend to rely on intuitive processing as a default, nodding along unless they have a reason or cue that triggers them to engage in more effortful, analytical processing (22). Verbatim repetition is easier for information consumers to recognize (21, 53), while the nonverbatim repetition that defines AIPasta may appear more authentic due to its lexical diversity (or perception of multiple, confirmatory information sources). This perceived authenticity means consumers encountering AIPasta may take longer to recognize it as a persuasive attempt and decrease the likelihood of them engaging in the more careful processing needed to identify it as false or misleading information. Compounding this impact is the fact that prior work shows that false information is harder to correct after exposure to nonverbatim repetition, as compared to verbatim repetition (54, 55). While content manually generated by humans may have similar advantages, the ability of AIPasta to generate such content at scale with minimal resources sets it apart as a new and unique threat to the spread of false and/or misleading information.

Interestingly, in the current study, participants perceive CopyPasta and AIPasta with similar intent to persuade; 5.14±0.08 and 5.20±0.07, respectively. The high ratings across both conditions (close to the maximum value of 6), however, could potentially be an artifact of the exaggerated number of repetitions in both treatments (7 out of 10 posts). Future research should examine whether different frequencies of repetitions lead to stronger or weaker effects of AIPasta (and CopyPasta).

The results in this study likely represent a lower bound of the potential impact of AI-driven false information due to its single exposure and shorter-term timeline for measuring effects. In reality, depending on the campaign strategy, individuals might be exposed to information over extended periods of time. As the effects of repeated exposure on belief are larger when those exposures are spaced across time rather than occurring all at once (56), this could result in stronger effects than those observed here, further amplifying the impact on public opinion and behavior. Together, these effects are likely to translate into wider spread and engagement with strategic information campaigns characterized by AIPasta. Future work should investigate long-term effects of such strategic information campaigns by conducting longitudinal experiments and/or using observational data.

The incorporation and subsequent evaluation of generative AI tools in strategic information campaigns represents a potentially significant leap in the effectiveness and efficiency of these operations and merits scholarly and public attention. By highlighting the role of LLMs in facilitating more sophisticated, low-detectability misleading information operations, this study contributes to the growing body of work aiming to better understand the potential threats of AI-generated information and its influence on people’s beliefs. LLMs evolve quickly, with new models and updates emerging frequently. By focusing on the underlying heuristic cues that LLMs can modify or enhance, we create a framework that remains relevant across different versions of LLMs. This approach and framework allow for continuous application and analysis throughout specific model changes.

Additionally, this study and future work can inform current interventions and offer opportunities for new designs to mitigate the spread and impact of harmful content. Insights can guide the development of platform strategies and policies, as well as community-driven efforts in education and outreach. These could include information literacy campaigns about the nature and impact of AI-generated misinformation and disinformation, as well as policies that promote digital safety.

## Materials and methods

### Hypotheses

In this study, we explore how generative AI may amplify the persuasive effects of disinformation campaigns by enhancing heuristic cues like repetition. We ground our study in the widely replicated, robust phenomenon of the illusory truth effect which states that repetition of a claim increases its perceived truth through increased cognitive fluency. Overall, we hypothesize that AIPasta can increase the perceived truth of disinformation campaign claims through the illusory truth effect (17), without increasing the perceptions of manipulation intent that traditional CopyPasta campaigns induce (through verbatim repetition). We measure perceived truth along three dimensions—belief in the exact claim viewed during message exposure, belief in the broader disinformation claim, and belief in a related disinformation claim (questions are detailed in outcome measures). Building from this perspective, we formulate and preregister the following hypotheses. Some of the language for hypotheses H1, H2, H8, and H9 differ from the preregistration; view the Open Science Statement for more details.

H1 (Perceived Truth; Repetition Vs Control): Participants exposed to repetitive messages (CopyPasta or AIPasta) are more likely to perceive the disinformation campaign claim (exact, broad, and related) as true than those who are not exposed to repetitive messages (Control).
H2 (Perceived Truth; CopyPasta Vs AIPasta): Participants exposed to AI-generated, nonverbatim repetitive messages (AIPasta) are more likely to perceive the disinformation campaign claim (exact, broad, and related) as true than those who are exposed to verbatim, repetitive messages (CopyPasta).
H3 (Perceived Intent to Persuade; CopyPasta Vs AIPasta): Participants exposed to AI-generated, nonverbatim repetitive content (AIPasta) are less likely to feel that messages are intended to persuade them as compared to those who are exposed to verbatim, repetitive messages (CopyPasta).

Furthermore, both verbatim (e.g. CopyPasta) and nonverbatim (e.g. AIPasta) repetition have been shown to increase the perception of social consensus (27). Hence, we hypothesize that:

H4 (Perceived Social Consensus; Repetition vs. Control): Participants exposed to repetitive messages (CopyPasta or AIPasta) are more likely to perceive that there is greater social consensus for the misinformation campaign claim as compared to those who are not exposed to repetitive messages (Control).

Additionally, we hypothesize differences in perceived social consensus between nonverbatim (AIPasta) and verbatim (CopyPasta) repetition. Reference (27) find similar increases in perceptions of social consensus for verbatim repetition from one source and nonverbatim repetition from multiple sources. However, our experiment differs from theirs as we maintain the same number of sources across verbatim and nonverbatim repetition. Hence, in this setting, AI-generated, nonverbatim repetitive messages (AIPasta) may increase perceptions of social consensus, compared to the effects of repetitive, verbatim messages (CopyPasta), or have no difference. Therefore, we evaluate which of the following may be supported:

H5a (Perceived Social Consensus; CopyPasta vs. AIPasta): Participants exposed to AI-generated, nonverbatim repetitive messages (AIPasta) will have increased perceptions of social consensus for the misinformation campaign claim as compared to those who are exposed to repetitive, verbatim messages (CopyPasta).
H5b (Perceived Social Consensus; CopyPasta vs. AIPasta): Participants exposed to AI-generated, nonverbatim repetitive messages (AIPasta) will have similar perceptions of social consensus for the misinformation campaign claim as compared to those who are exposed to repetitive, verbatim messages (CopyPasta).

Moreover, repetition has been shown to increase the ease of recall of pertinent information in individuals, which has further downstream effects on attitudinal changes (57). According to the perceptual fluency explanation for repetition, verbatim repetition should lead to greater increases in processing fluency of the campaign claim, compared to nonverbatim repetition. However, according to the conceptual fluency explanation for repetition, both verbatim and nonverbatim repetition should have similar increases in processing fluency (25, 58). Therefore, we predict that:

H6 (Recall; Repetition vs. Control): Participants exposed to repetitive messages (CopyPasta or AIPasta) are more likely to recall the misinformation campaign claim than those who are not exposed to repetitive messages (Control).
H7a (Recall; CopyPasta vs. AIPasta): Participants exposed to AI-generated, nonverbatim repetitive messages (AIPasta) are less likely to recall the misinformation campaign claim as compared to those who are exposed to verbatim, repetitive messages (CopyPasta).
H7b (Recall; CopyPasta vs. AIPasta): Participants exposed to AI-generated, nonverbatim repetitive messages (AIPasta) are equally likely to recall the misinformation campaign claim as compared to those who are exposed to verbatim, repetitive messages (CopyPasta).

Finally, repetition of misinformation has been linked to increased intention of sharing the misinformation online (37). Therefore, we also hypothesize differences in the sharing behavior of participants exposed to repetitive messaging, as compared to control.

H8 (Sharing Intention; Repetition vs. Control): Participants exposed to repetitive messages (CopyPasta or AIPasta) are more likely to share the disinformation campaign claim (exact, broad, and related) than those who are not exposed to repetitive messages (Control).

Additionally, Ref. (37) finds that the perceived truth of the misinformation claim moderates sharing intention. Hence, if H2 is true, we expect the increased perceived truth of AIPasta to be linked to increased sharing intent as compared to CopyPasta.

H9 (Sharing Intention; CopyPasta vs. AIPasta): Participants exposed to AI-generated, nonverbatim repetitive messages (AIPasta) are more likely to share the disinformation campaign claim (exact, broad, and related) as compared to those who are exposed to verbatim, repetitive messages (CopyPasta).

### Dataset

In this study, we utilize two social media datasets collected by the Center for an Informed Public at the University of Washington. Each dataset was originally collected using a set of keyword-based queries to sample content from the Twitter (now X) API, in a strategy similar to that described in (59) and (60), #StopTheSteal posts were collected querying for election-related keywords, while the #Plandemic posts were collected using coronavirus and pandemic terms. We then filter for tweets that at least contain the hashtag of interest for the disinformation campaigns. The dataset consists of tweets posted between 2020 September 1 to December 15. The #StopTheSteal campaign was prominent after the elections in the United States on 2020 November 4 , and the #Plandemic campaign gained traction after the documentary Plandemic video was released on 2020 May 4 (61) (our study was conducted between 2024 May 31 and June 1). While the resulting datasets may not be comprehensive, completeness is not necessary for this work where we simply need representative messages from the CopyPasta campaigns to assess their lexical features and use as inputs to the AIPasta model. We begin with a dataset of 342,054 posts containing #StopTheSteal and 347,180 posts containing #Plandemic. We also note that sampling data in this way is no longer possible within reasonable resource constraints due to recent changes in the X API.

### Identifying CopyPasta campaign tweets

After extracting campaign-related tweets in the data via hashtags, we identify CopyPasta campaigns by following the methodology for coordination detection in Ref. (15). Briefly, we consider original tweets in the dataset (not including retweets) and extract high-dimensional tweet embeddings using an extended version of the multilingual Universal Sentence Encoder (mUSE) (62). We construct a network among tweets where edges represent similarity, adding a weighted edge between two tweets where the weight is the pairwise cosine similarity. We filter out edges with a similarity lesser than 0.9 in order to retain a network of tweets that are highly similar to each other. We use Louvain’s community detection algorithm to identify groupings of tweets that were used in distinct CopyPasta campaigns. This procedure identified 14,213 and 11,905 campaign tweets in the #StopTheSteal and #Plandemic case studies, respectively. When considering campaigns of at least five posts, we identify 2,276 and 1,563 tweets in the #StopTheSteal and #Plandemic case studies, respectively.

### AIPasta generation

To generate AIPasta, we use ChatGPT with temperature 0.7 to paraphrase CopyPasta tweets according to the following rules (25): (i) the paraphrased tweet should have approximately the same number of words as the original tweet, (ii) the paraphrased tweet should contain as few words as possible from the original tweet by substituting synonyms of appropriate words, (iii) the structure of the original tweet should be changed by reordering elements of the tweet, and (iv) the semantic meaning of the paraphrased and original tweet should remain the same. The exact prompt to the LLM is

Paraphrase the tweet inputted by the user by following these rules:

1. The paraphrased tweet should have approximately the same number of words as the original tweet.
2. Repeat as few words as possible from the original tweet by substituting synonyms of appropriate words. Do not substitute hashtags or common phrases. Use simple words as synonym substitutions.
3. Change the structure of the original tweet by reordering elements of the tweet.
4. Ensure that the meaning of the original tweet and the paraphrased tweet are the same. Paraphrase the original tweet 10 times. Make sure the language is informal and easy to read.

Below we provide examples of the input/output pairs for this procedure (see Supplementary Information for example Stimuli). Input Post (Identified CopyPasta):

*Remember the vaccine remains substantially more dangerous than the virus! Adverse reactions include Thrombosis, Embolism, Stroke, Heart attack, blindness and death! You have been warned!*

Output posts (3 out of 10 generated AIPasta displayed, refer to registration for all generated data):

*Just a heads up, the vaccine is actually riskier than the virus! It can cause serious issues like blood clots, strokes, heart attacks, blindness, and even death. Be careful!*
*Remember, the vaccine is more dangerous than the virus! Watch out for side effects like blood clots, strokes, heart attacks, blindness, and death. Stay cautious!*
*Heads up, the vaccine is more unsafe than the virus! Adverse reactions can lead to blood clots, strokes, heart attacks, blindness, and death. Take care!*

### AIPasta validation metrics

*Semantic similarity.* The cosine similarity between two vector embeddings A and B is:


$$\text{cosine}\_\mathrm{similarity}(A,B)=\;\frac{A\cdot B}{\left\|A\|\;\|B\right\|}$$

*Lexical diversity.* Measured by the type-token ratio (TTR), which is the ratio of the number of unique words (types, V) to the total number of words (tokens, *N*) in a text:


$$\text{TTR}=\;\frac{V}{N}$$

### Participants and procedure

A sample of US participants was recruited via Prolific (an online crowdsourcing platform), representative of age, sex, and political affiliation in the United States.^e^, using its prescreening criteria. Participants were paid $1.20 for completing the survey (for which the median time was ∼8 min, 43 s). Participants had to be residing in the United States and at least 18 years old to participate. There were 1,200 participants in total, with n=401, and 405 in AIPasta and CopyPasta, respectively. The sample composition of participants is displayed in Table 2. Although we preregistered to exclude participants who spent <5 s on the stimuli (only seven participants met this criterion in our sample), we decided to retain all participant responses in the analysis because the chosen threshold was arbitrary (informed by prior message effects studies that often assume longer reading times) and the empirical data showed distributed response times without an obvious cutoff. We also recognize that brief exposure is typical in real-world social media use and does not necessarily indicate inattention. Therefore, we determined that retaining these few participants would preserve ecological validity and better reflect natural browsing behavior. Additionally, robustness checks confirmed that excluding these participants did not alter the direction or significance of our findings.

**Table 2.** Demographic summary.

	*N*	%
Sex		
Female	612	51.0
Male	588	49.0
Age		
18–24	140	11.67
25–34	210	17.50
35–44	202	16.83
45–54	190	15.83
55 or older	458	38.16%
Political affiliation		
Independent	514	42.83
Republican	355	29.58
Democrats	331	27.58

A sample of US participants was recruited using Prolific, representative of age, sex, and political affiliation in the United States, using its prescreening criteria.

The study used a three-condition (CopyPasta, AIPasta, or Control) between-subjects design, with each participant making ratings on two topics (#StopTheSteal and #Plandemic, randomly ordered) to reduce item effects. After reading the consent form, participants answered a series of randomly ordered questions assessing their preexisting familiarity with the disinformation narratives along with some filler true narratives. Next, participants were given the instructions “You will be shown some posts from a real social media feed now. Take some time to read them. Afterwards, you will be asked some follow-up questions regarding the posts..” They were then directed to the first stimuli page to view a series of posts. These posts corresponded to one of the two research topics (randomly selected), and the order the posts appeared on the page was randomized for each participant.

**Fig. 7.**
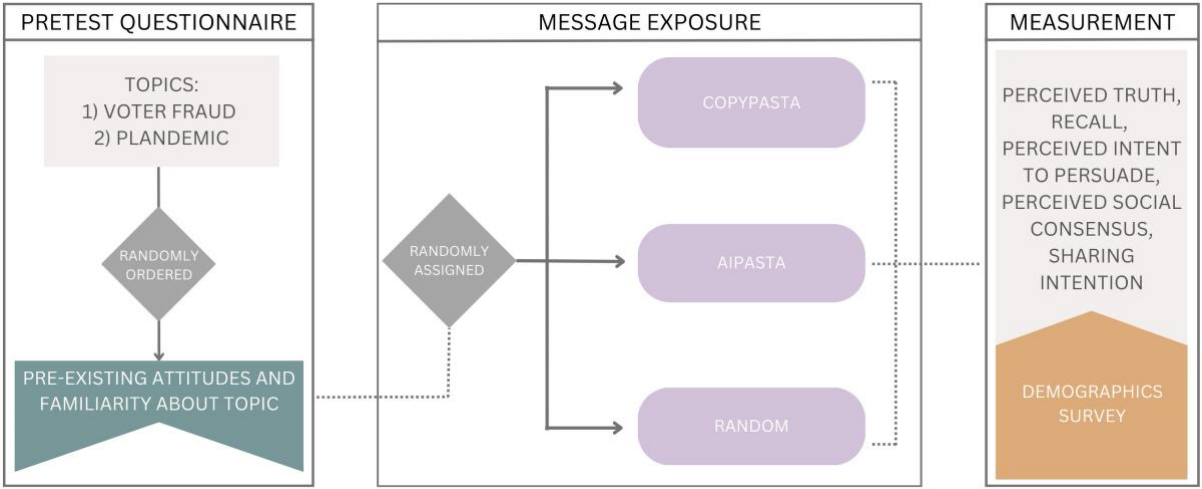
Survey flow. The study used a three-condition (CopyPasta, AIPasta, and Control) between-subject design, with each participants making ratings on two topics (#StopTheSteal and #Plandemic) to reduce item effects.

Immediately after, participants were asked to evaluate the perceived truthfulness of the messaging (exact false post, the broad false narrative claim, and the related claim), complete the recall question, rate the perceived intent of the posts to persuade, and the perceived social consensus and finally judge their likelihood of sharing an exact post. Participants were then directed to the next stimuli page (on the second research topic) and answered the same questions. The two research topics were presented randomly to avoid order effects. At the end, participants answered a series of demographic questions. Participants finished the survey with a debrief in which each false or misleading information claim was addressed explicitly and multiple references were provided to support the position that these claims are false or misleading. The survey flow is depicted in Fig. 7.

### Analytic approach

In order to test hypotheses H1, H4, H6, and H8, we use a linear mixed-effects model for each outcome measure. We dummy code the treatment variables of CopyPasta and AIPasta, contrasting against the Control condition (which serves as the reference category) while controlling for the Topic. Significantly positive coefficients for CopyPasta and AIPasta treatments are interpreted as evidence in favor of the hypotheses.

To test hypotheses H2, H3, H5, H7, and H9, we also use a linear mixed-effects model for each outcome measure. We dummy code the treatment variable of AIPasta and Control condition, contrasting against the CopyPasta condition (which serves as the reference category) while controlling for the Topic. A significantly positive coefficient for the AIPasta treatment is interpreted as evidence in favor of hypothesis H2. A significantly negative coefficient for AIPasta is treated as evidence for H3, while a significantly positive coefficient for H5a, or a nonsignificant result for H5b is treated as evidence in favor of the hypothesis. A significantly negative coefficient for H7a or nonsignificant result for H7b is treated as evidence in favor of the hypothesis. Finally, a significantly positive coefficient for H9 is treated as evidence for the hypothesis.

In all models, the fixed effects include the treatment conditions of repetition (AIPasta, CopyPasta) and topic, while the random effects consist of participant IDs, accounting for the correlated nature of repeated measures within the same participant (due to exposure to both topics). We use one linear mixed model for each of the outcome variables, except for Perceived Truth (Exact Claim) and Sharing Intention, where half of the participants in the control condition rate a randomly selected stimulus of AIPasta, and the other half of control condition rate CopyPasta. For these outcomes, we split the Control into two groups and use two linear mixed models, one for AIPasta contrasted against the Control participants who rate AIPasta posts and one for CopyPasta contrasted against the Control participants who rate CopyPasta posts. Doing so allows us to isolate the effects of repetition from baseline differences in ratings between AIPasta and CopyPasta (i.e. item effects). We note that while this portion of the analysis was not preregistered, we ultimately decided that splitting the control in this manner was a more appropriate analysis for testing our hypotheses related to these outcomes.

Finally, for our exploratory analyses testing whether partisanship and familiarity moderated the effects of the treatment conditions on the outcome variables by creating interaction terms between the dummy coded treatment variables and party affiliation/familiarity. For familiarity specifically, we probed significant interactions using the Johnson–Neyman technique to test whether the different treatments of AIPasta/CopyPasta had varying effects with differing levels of familiarity on the outcome variables.

### Limitations

It is important to note that this study has several limitations. One limitation of this study is the selection of repetition frequency when constructing stimuli for verbatim and nonverbatim repetition. There are multiple options regarding the number of repetitions and the inclusion of fillers. We chose a frequency that closely resembles real-world CopyPasta campaigns. However, because the effects of repetition on belief and perceived persuasion varies depending on the number of repetitions (e.g. (21, 34)) future research should explore varying repetition frequencies and investigate whether conditional effects arise based on the number of repetitions in the stimuli. Another limitation concerns the selection of research topics. We chose two influential real-world disinformation campaigns to enhance the ecological validity of the study. However, if participants were already highly familiar with these campaigns, it could have diluted the observed effects. To partially address this, we measured participants’ familiarity and included it as a variable in our analysis. Future research could study topics that participants are less familiar with to see whether similar effects are observed. Furthermore, participants exhibited a greater likelihood of recalling the stimuli presented in AIPasta compared to those in CopyPasta. However, the lower similarity scores observed for CopyPasta may be partially attributed to the fact that some responses merely noted that the messages were copied and pasted, without further elaborating on the content of the messages.

**IRB:** The study design and materials were reviewed by the Human Subjects Division at the University of Washington and determined to be human subjects research that qualifies for exempt status (category 3). This determination may or may not be based on the Limited IRB Review process. Informed consent was obtained from all participants prior to participation.

## Supplementary Material

pgaf207_Supplementary_Data

## Data Availability

Data, code and materials are available at https://osf.io/jzdkf/.

## Notes

a
https://osf.io/rpu7c/?view_only=89c35e76a48543d986f86da859a6c3d6

b
https://openai.com/index/disrupting-deceptive-uses-of-AI-by-covert-influence-operations/

c
https://help.x.com/en/rules-and-policies/copypasta-duplicate-content

d
https://huggingface.co/spaces/evaluate-metric/perplexity

e
https://researcher-help.prolific.com/en/article/e6555f

## Preprints

A preprint for this manuscript was posted at https://osf.io/preprints/osf/8zb5p_v1.
