# Supplementary material for: The persuasive potential of AI-paraphrased information at scale
Source: PNAS Nexus. 2025 Jul 22;4(7):pgaf207. doi: 10.1093/pnasnexus/pgaf207 (PMC12281505; doi:10.1093/pnasnexus/pgaf207)
Supplement: pgaf207_Supplementary_Data [file pgaf207_supplementary_data.pdf]

Supplementary Information

Stimuli

To select tweets from CopyPasta campaigns as input for generating AIPasta, we look at the biggest (top 100) CopyPasta campaigns (identified strategy described above). We ignore tweets with links (to avoid participants being redirected to other sites), tweets with only hashtags, call-to-action tweets, and news/podcast headlines, focusing instead on text-rich posts related to the campaign narrative. We filter out tweets with a Flesch reading score [1, 11] between 60-69 (on a scale of 1-100 and interpreted as approximately grade 8 reading level).

To generate the AIPasta used as stimuli, we leverage human-machine teaming to select the best AIPasta tweets. We had one of the authors go through the generated AIPasta tweets and select tweets that were paraphrased according to the instructions provided to the LLM. Similar to [7], we intentionally had only one author perform this task in order to closely mimic a realistic scenario of how disinformation operations may be organized.

The generated text was then edited into tweet form, using profile images, usernames, and handles constructed by [9]. These profiles did not represent real users and were created from stock images and with handles that were not currently in use. Both AIPasta and CopyPasta conditions had 7 AI/CopyPasta tweets and 3 filler tweets. The filler tweets were created using gpt-3.5-turbo [16].

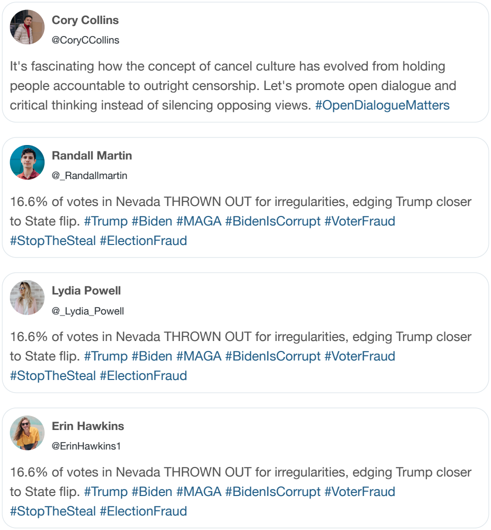

Fig. S1. #StopTheSteal CopyPasta Stimuli

Survey Questions

The following questions were asked in the survey. For each, we provide the survey prompt and response options.

Familiarity
Please indicate your familiarity with the following statements (based on how much you have heard about the topic). (6 point Likert scale ranging from Not familiar - Extremely familiar)
1) There was widespread voter fraud in the 2020 U.S. Presidential elections. (Not familiar - Extremely familiar)

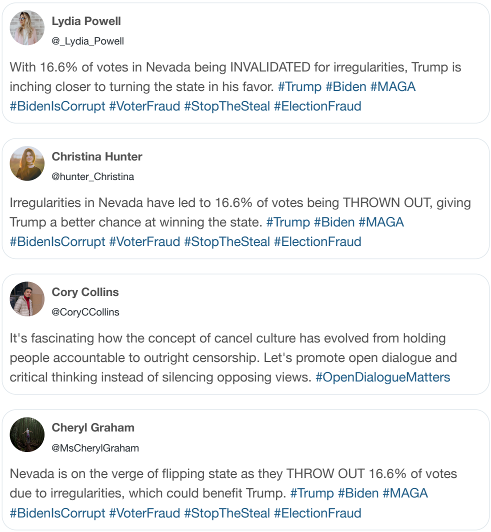

Fig. S2. #StopTheSteal AIPasta Stimuli

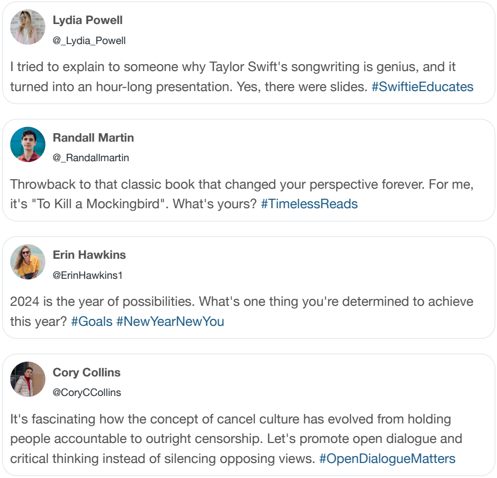

Fig. S3. #StopTheSteal Control Stimuli

2) The coronavirus pandemic was planned by powerful groups. (Not familiar - Extremely familiar)
3) Human activities are the main drivers of global climate change. (Not familiar - Extremely familiar)
4) MMR vaccines cause Autism Spectrum Disorder (ASD). (Not familiar - Extremely familiar)
5) The risk of death associated with childbirth is higher than that with abortion. (Not familiar - Extremely familiar)
6) Immigrants are a major source of crime in the U.S. (Not familiar - Extremely familiar)
7) GMO (genetically modified organism) foods are as healthy and safe to eat as their non-GMO counterparts. (Not familiar - Extremely familiar)

Perceived Truth
Participants in the AIPasta/CopyPasta conditions saw a randomly selected AIPasta/CopyPasta post that they were previously exposed to, while participants in control saw a randomly selected post from the pool of all AIPasta and

CopyPasta stimuli for that topic and asked the following question for the Perceived Truth (Exact Claim) measurement:
How likely do you think it is that the information contained in this is post true? (6 point Likert scale ranging from Extremely unlikely - Extremely Likely) (*Exact Claim* for both Topics)

Broadly, do you agree or disagree that votes cast by absentee or mail-in ballot were not counted as voters intended in the 2020 U.S. election? (6 point Likert scale ranging from Strongly disagree - Strongly agree) (*Related Claim* for #StopTheSteal) [4]
How likely do you think it is that there was widespread voter fraud (that is people who were not eligible voted, or people cast multiple ballots) in the 2020 U.S. election? (6 point Likert scale ranging from Extremely unlikely - Extremely Likely) (*Broader Claim* for #StopTheSteal) [2]

Broadly, do you agree or disagree that the seriousness of coronavirus was generally exaggerated? (6 point Likert scale ranging from Strongly disagree - Strongly agree) (*Related Claim* for #Plandemic) [10]
How likely do you think it is that the coronavirus pandemic was planned by a group of powerful people? (6-point Likert scale ranging from Extremely unlikely - Extremely Likely) (*Broader Claim* for #Plandemic) [3]

Recall
What do you think most of the posts were talking about? Be as specific as you can (at least 30 characters).

Perceived Intent to Persuade
In general, while reading the tweets, did you feel like the authors of the tweets were trying to convince you of their views? (6 point Likert scale ranging from Definitely not - Definitely yes) [12]

Perceived Social Consensus
To the best of your knowledge, what percentage of people in the U.S. support the claim that there was widespread voter fraud during the 2020 elections? (0-100) [6]
To the best of your knowledge, what percentage of people in the U.S. support the claim that the coronavirus outbreak was planned by powerful people? (0-100) [6]

Sharing Intent
Participants in the AIPasta/CopyPasta conditions saw a randomly selected AIPasta/CopyPasta post that they were previously exposed to, while participants in control saw a randomly selected post from the pool of all AIPasta and CopyPasta stimuli for that topic and were asked the following question for measuring Sharing Intent:
How likely would you be to share this post online? (6-point Likert scale ranging from Extremely unlikely - Extremely Likely)

Demographic Information
What is your age?

What is the highest level of school you completed, or the highest degree you earned?

- Some high school or less
- High school diploma or GED
- Some college, but no degree
- Associates or technical degree
- Bachelor's degree
- Graduate or professional degree (MA, MS, MBA, PhD, JD, MD, DDS etc.)
- Prefer not to say

Which of the following best describes your race? Check all that apply.

- White or Caucasian
- Black or African American
- American Indian/Native American or Alaska Native
- Asian
- Native Hawaiian or Other Pacific Islander
- Other
- Prefer not to say

Generally speaking, do you think of yourself as a...

- Republican
- Democrat
- Independent
- Other
- No preference

[If Independent or Other or No preference is selected] Do you think of yourself as closer to the Republican or Democratic party?

- Republican Party
- Democratic Party

Recall

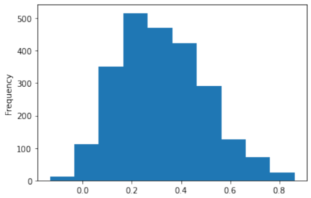

Fig. S4. Distribution of Cosine Similarities Between Recall Responses and Stimuli

Participants exposed to AIPasta messaging (b = 0.213, CI = [0.199, 0.226], $p < 0.001$) or CopyPasta (b = 0.175, CI = [0.162, 0.189], $p < 0.001$) reproduce information more similar to the posts they had seen compared to the control condition when asked what they thought most of the posts were talking about, supporting H6. Additionally, participants exposed to AIPasta are significantly more likely to recall the false claims as compared to those in the CopyPasta condition (b = 0.037, CI = [0.024, 0.051], $p < 0.001$), supporting H7B (that recall would be better for AIPasta vs. CopyPasta) over H7A (that recall would be similar for both).

AIPasta was also more likely to be recalled compared to CopyPasta. This may be attributed to the fact that AIPasta, with its non-verbatim content, is inherently more engaging and easier to process and encode than the repetitive nature of CopyPasta. Research suggests that when individuals engage with more interesting and engaging content, they utilize fewer attentional resources for comprehension [14]. Future research should investigate whether such immediate recall can

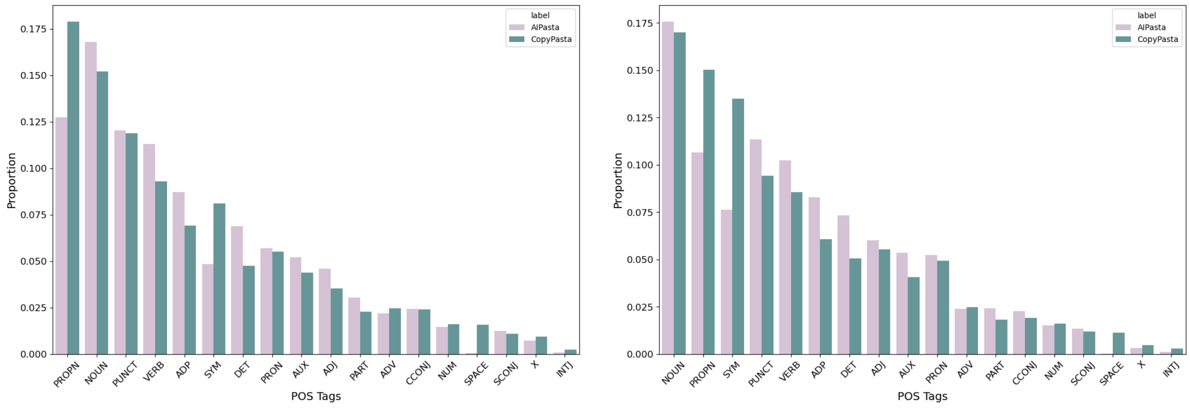

(a) #StopTheSteal (b) #Plandemic

Fig. S5. POS Tagging. Linguistic features analysis through POS tagging for the AIPasta and CopyPasta conditions across the two topics.

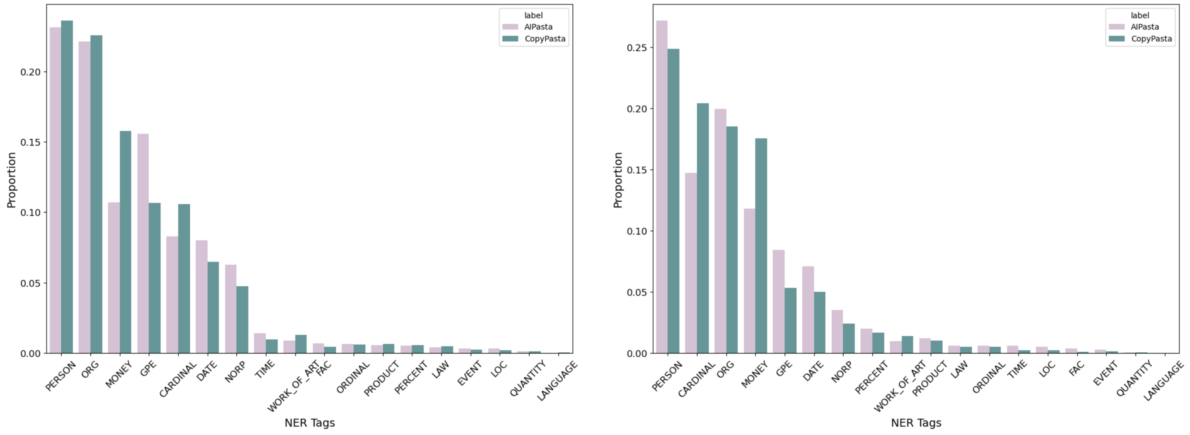

(a) #StopTheSteal (b) #Plandemic

Fig. S6. Named Entity Tags. Analysis of named entity recognition (NER) for the AIPasta and CopyPasta conditions across the two topics.

also enhance long-term (mis)information retention to better understand the broader implications of increased recall of AIPasta.

Recall Analysis

The recall responses by participants are automatically scored for similarity between the misinformation stimuli displayed to them and their response text. To do this, we compute the cosine similarity of the mUSE embeddings of the stimuli and the response text. The distribution of the cosine similarities is given in Figure S4.

AI-Text Detectors

To understand whether AI-Paraphrased text is identified as AI-generated text, we use a suite of open-source AI-generated text detectors. GLTR [5] and PPL [8] are zero-shot approaches that rely on the likelihood outputs of a pretrained language model, while the OpenAI detector [18] is a RoBERTa-based classifier trained on GPT-2's [17] training corpus. RoBERTa-MPU [19] is also a RoBERTa based classifier that uses a Multiscale Positive-Unlabeled (MPU) loss to boost performance on short corpora. MAGE [13] is a longformer-based text detector trained on a diverse corpus of 447K human-written and machine-generated texts in the wild. We exclude detectors like DetectGPT [15], because of their white-box assumption, which requires access to the AI model's prediction distributions.

References

1. Rudolph Flesch. A new readability yardstick. *Journal of applied psychology*, 32(3):221, 1948.
2. Center for Democracy, University of Maryland Civic Engagement, and Washington Post. Washington post-university of maryland poll, question 16 [31120703.00015], 2023.
3. Pew Research Center for the People & the Press. Pew research center: American trends panel wave 68, question 47 [31117491.00076], 2020.
4. Pew Research Center for the People & the Press. Pew research center: American trends panel wave 75, question 61 [31118005.00060], 2020.
5. Sebastian Gehrmann, Hendrik Strobelt, and Alexander M Rush. Gltr: Statistical detection and visualization of generated text. *arXiv preprint arXiv:1906.04043*, 2019.
6. Matthew H Goldberg, Sander van der Linden, Anthony Leiserowitz, and Edward Maibach. Perceived social consensus can reduce ideological biases on climate change. *Environment and Behavior*, 52(5):495–517, 2020.

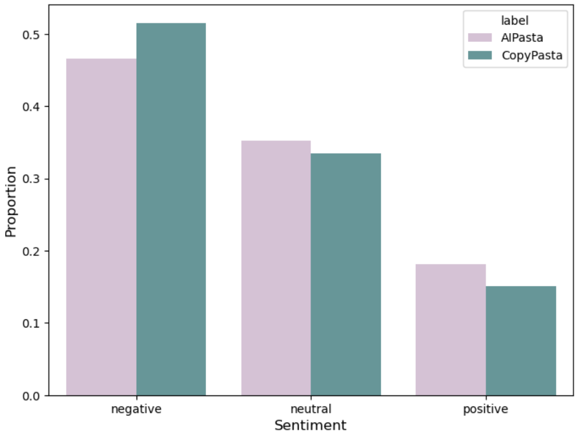

(a) #StopTheSteal

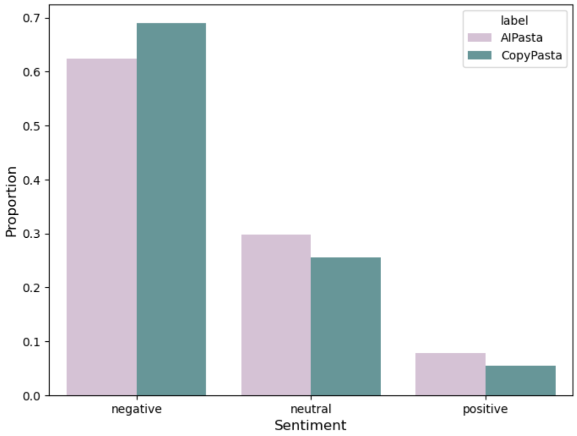

(b) #Plandemic

Fig. S7. Sentiment Analysis. Sentiment polarity results for the AIPasta and CopyPasta conditions across the two topics.

Detector	CopyPasta Recall	AIPasta Recall	Avg Recall
GLTR [5]	48.85%	51.88%	50.37%
MAGE [13]	59.08%	52.02%	55.55%
RpBERTa-MPU [19]	99.27%	3.18%	51.22%
PPL [8]	99.40%	17.52%	**58.46%**
OpenAI [18]	61.97%	43.41%	52.69%

Table S1. AIPasta Detection (#Plandemic): Similar to the #StopTheSteal AIPasta results, off-the-shelf AI-generated text detectors perform poorly at detecting AIPasta as AI-generated. Most models perform only marginally better than random.

Dependent Variable	Estimate	Std Err	P-Value	CI [0.025, 0.975]
**Perceived Truth (Exact Claim)**				
AIPasta Vs Control *(H1)*	0.133	0.079	0.091†	[-0.021, 0.287]
Copypasta Vs Control *(H1)*	0.006	0.081	0.939	[-0.152, 0.164]
AIPasta Vs CopyPasta *(H2)*	0.109	0.065	0.095†	[-0.019, 0.237]
**Perceived Truth (Broad Claim)**				
AIPasta Vs Control *(H1)*	0.020	0.067	0.770	[-0.112, 0.151]
Copypasta Vs Control *(H1)*	0.079	0.067	0.237	[-0.052, 0.211]
AIPasta Vs CopyPasta *(H2)*	-0.060	0.067	0.375	[-0.192, 0.072]
**Perceived Truth (Related Claim)**				
AIPasta Vs Control *(H1)*	0.049	0.066	0.460	[-0.081, 0.179]
Copypasta Vs Control *(H1)*	0.129	0.066	0.051†	[0.000, 0.259]
AIPasta Vs CopyPasta *(H2)*	-0.080	0.066	0.227	[-0.211, 0.050]
**Perceived Intent To Persuade**				
AIPasta Vs Control *(H3)*	1.214	0.051	< 0.001***	[1.114, 1.315]
Copypasta Vs Control *(H3)*	1.144	0.051	< 0.001***	[1.044, 1.244]
AIPasta Vs CopyPasta *(H3)*	0.070	0.051	0.170	[-0.030, 0.171]
**Perceived Social Consensus**				
AIPasta Vs Control *(H4)*	1.727	0.756	0.022*	[0.246, 3.208]
Copypasta Vs Control *(H4)*	1.200	0.755	0.112	[-0.281, 2.680]
AIPasta Vs CopyPasta *(H5a, H5b)*	0.527	0.759	0.487	[-0.960, 2.015]
**Recall**				
AIPasta Vs Control *(H6)*	0.213	0.007	< 0.001***	[0.199, 0.226]
Copypasta Vs Control *(H6)*	0.175	0.007	< 0.001***	[0.162, 0.189]
AIPasta Vs CopyPasta *(H6a, H6b)*	0.037	0.007	< 0.001***	[0.024, 0.051]
**Sharing Intention**				
AIPasta Vs Control *(H8)*	-0.055	0.058	0.341	[-0.168, 0.058]
Copypasta Vs Control *(H8)*	-0.187	0.057	< 0.001**	[-0.299, -0.075]
AIPasta Vs CopyPasta *(H9)*	0.083	0.047	0.075†	[-0.008, 0.175]

Table S2. Linear Mixed Model Coefficient Estimates with 95% CI. Models evaluate the impact of experimental conditions on outcomes of interest.†- $p < 0.1$, * - $p < 0.05$, ** - $p < 0.01$, *** - $p < 0.001$

Dependent Variable	Estimate	Std Err	P-Value	CI [0.025, 0.975]
**Perceived Truth (Exact Claim)**				
(AIPasta Vs Control) x Party	0.304	0.152	0.046*	[0.005, 0.603]
(Copypasta Vs Control) x Party	0.155	0.155	0.318	[-0.149, 0.460]
(AIPasta Vs CopyPasta) x Party	0.109	0.126	0.387	[-0.138, 0.356]
**Perceived Truth (Related Claim)**				
(AIPasta Vs Control) x Party	0.054	0.128	0.675	[-0.198, 0.305]
(Copypasta Vs Control) x Party	-0.015	0.128	0.907	[-0.265, 0.236]
(AIPasta Vs CopyPasta) x Party	0.069	0.128	0.592	[-0.183, 0.320]
**Perceived Intent to Persuade**				
(AIPasta Vs Control) x Party	-0.438	0.103	< 0.001***	[-0.640, -0.236]
(Copypasta Vs Control) x Party	-0.396	0.103	< 0.001***	[-0.596, -0.195]
(AIPasta Vs CopyPasta) x Party	-0.042	0.103	0.679	[-0.244, 0.159]
**Perceived Social Consensus**				
(AIPasta Vs Control) x Party	3.395	1.519	0.026*	[0.420, 6.370]
(Copypasta Vs Control) x Party	0.838	1.513	0.580	[-0.070, 0.422]
(AIPasta Vs CopyPasta) x Party	2.557	1.518	0.092†	[-3.800, 2.125]
**Recall**				
(AIPasta Vs Control) x Party	0.025	0.014	0.078†	[-0.003, 0.052]
(Copypasta Vs Control) x Party	0.010	0.014	0.477	[-0.017, 0.037]
(AIPasta Vs CopyPasta) x Party	0.015	0.014	0.289	[-0.013, 0.042]
**Sharing Intention**				
(AIPasta Vs Control) x Party	0.133	0.116	0.252	[-0.094, 0.360]
(Copypasta Vs Control) x Party	-0.163	0.114	0.152	[-0.387, 0.060]
(AIPasta Vs CopyPasta) x Party	0.169	0.093	0.069†	[-0.013, 0.352]

Table S3. Linear Mixed Model Coefficient Estimates for Party Interaction Effects. Models evaluate the impact of experimental conditions interacting with party affiliation on outcomes of interest.†- $p < 0.1$, * - $p < 0.05$, ** - $p < 0.01$, *** - $p < 0.001$

Dependent Variable	Estimate	Std Err	P-Value	CI [0.025, 0.975]
**Perceived Social Consensus**				
(AIPasta Vs Control) x Familiarity	-1.043	0.458	0.023*	[-1.94, -0.147]
(Copypasta Vs Control) x Familiarity	-0.856	0.450	0.057†	[-0.070, 0.422]
(AIPasta Vs CopyPasta) x Familiarity	-0.186	0.450	0.676	[-1.059, 0.687]
**Recall**				
(AIPasta Vs Control) x Familiarity	0.012	0.004	0.005**	[0.004, 0.021]
(Copypasta Vs Control) x Familiarity	0.017	0.004	< 0.001***	[0.008, 0.025]
(AIPasta Vs CopyPasta) x Familiarity	-0.005	0.004	0.270	[-0.012, 0.004]

Table S4. Linear Mixed Model Coefficient Estimates for Familiarity Interaction Effects. Models evaluate the impact of experimental conditions interacting with topic familiarity on outcomes of interest.†- $p < 0.1$, * - $p < 0.05$, ** - $p < 0.01$, *** - $p < 0.001$)

7. Josh A Goldstein, Girish Sastry, Micah Musser, Renee DiResta, Matthew Gentzel, and Katerina Sedova. Generative language models and automated influence operations: Emerging threats and potential mitigations. *arXiv preprint arXiv:2301.04246*, 2023.
8. Biyang Guo, Xin Zhang, Ziyuan Wang, Minqi Jiang, Jinran Nie, Yuxuan Ding, Jianwei Yue, and Yupeng Wu. How close is chatgpt to human experts? comparison corpus, evaluation, and detection. *arXiv preprint arXiv:2301.07597*, 2023.
9. Madeline Jalbert, Mallory Harris, and Luke Williams. Who is perceived to be an expert on covid-19 vaccines on social media? biomedical credentials confer expertise, even among vaccine-hesitant and conservative observers. *Information, Communication & Society*, pages 1–19, 2025.
10. KFF. Kff poll: September 2023 covid-19 vaccine monitor, question 37 [31120562.00036], 2023.
11. JP Kincaid. Derivation of new readability formulas (automated readability index, fog count, and flesch reading ease formula) for navy enlisted personnel. 1975.
12. Thomas Koch and Thomas Zerback. Helpful or harmful? how frequent repetition affects perceived statement credibility. *Journal of Communication*, 63(6):993–1010, 2013.
13. Yafu Li, Qintong Li, Leyang Cui, Wei Bi, Zhilin Wang, Longyue Wang, Linyi Yang, Shuming Shi, and Yue Zhang. Mage: Machine-generated text detection in the wild. In *Proceedings of the 62nd Annual Meeting of*

the Association for Computational Linguistics (Volume 1: Long Papers), pages 36–53, 2024.

14. Mark A McDaniel, Paula J Waddill, Kraig Finstad, and Tammy Bourg. The effects of text-based interest on attention and recall. *Journal of educational psychology*, 92(3):492, 2000.
15. Eric Mitchell, Yoonho Lee, Alexander Khazatsky, Christopher D Manning, and Chelsea Finn. Detectgpt: Zero-shot machine-generated text detection using probability curvature. In *International Conference on Machine Learning*, pages 24950–24962. PMLR, 2023.
16. OpenAI. Gpt-3.5-turbo, 2023. Large Language model developed by OpenAI. Available at `https://platform.openai.com/docs/models/gpt-3-5`.
17. Alec Radford, Jeffrey Wu, Rewon Child, David Luan, Dario Amodei, Ilya Sutskever, et al. Language models are unsupervised multitask learners. *OpenAI blog*, 1(8):9, 2019.
18. Irene Solaiman, Miles Brundage, Jack Clark, Amanda Askell, Ariel Herbert-Voss, Jeff Wu, Alec Radford, Gretchen Krueger, Jong Wook Kim, Sarah Kreps, et al. Release strategies and the social impacts of language models. *arXiv preprint arXiv:1908.09203*, 2019.
19. Yuchuan Tian, Hanting Chen, Xutao Wang, Zheyuan Bai, Qinghua Zhang, Ruifeng Li, Chao Xu, and Yunhe Wang. Multiscale positive-unlabeled detection of ai-generated texts. *arXiv preprint arXiv:2305.18149*, 2023.
